# Supplementary material for: “I don’t see a reason why we should be hidden from view”: Views of a convenience sample of people living with HIV on sharing HIV status data in routinely collected health and care databases in England
Source: PLoS One. 2025 Feb 12;20(2):e0316848. doi: 10.1371/journal.pone.0316848 (PMC11819594; doi:10.1371/journal.pone.0316848)
Supplement: S1 File — (PDF) [file pone.0316848.s001.pdf]

THE LAW

Use of personal patient data outside of clinical environments is governed by:

- Data Protection Act (DPA) 2018 (also known as GDPR)
- Common Law Duty of Confidentiality (CLDC)
- Health and Social Care Act (2012, updated 2022)

'Personal data' means any information relating to an identified or identifiable natural person ('data subject'); an identifiable natural person is one who can be identified, directly or indirectly, in particular by reference to an identifier such as a name, an identification number, location data, an online identifier or to one or more factors specific to the physical, physiological, genetic, mental, economic, cultural or social identity of that natural person.

1

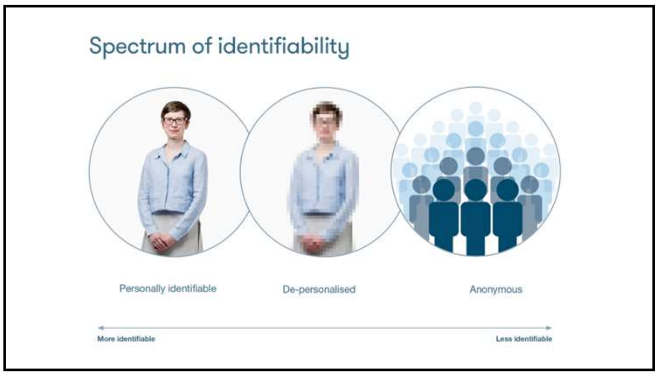

2

WHY NOT JUST ASK FOR CONSENT FOR USING PATIENT DATA?

There are several reasons why an **opt-out** approach may be preferable to **opt-in**:

- Although consent increases autonomy, it places a higher burden on patients
- An Opt-in system may introduce bias by only including a small portion of the population
- Who would opt in? Who would be left out?
- Underserved and seldom heard populations might be missing from the data.

3

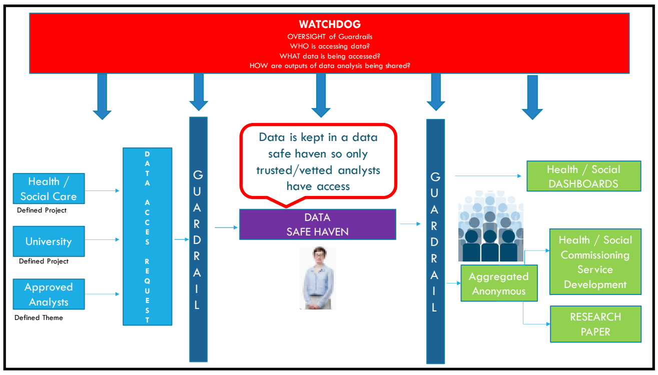

4

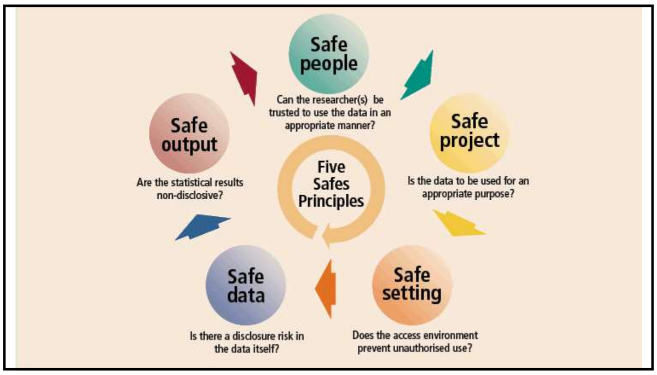

5

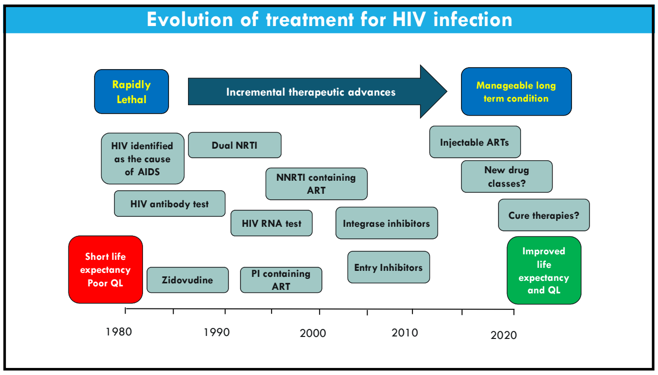

6

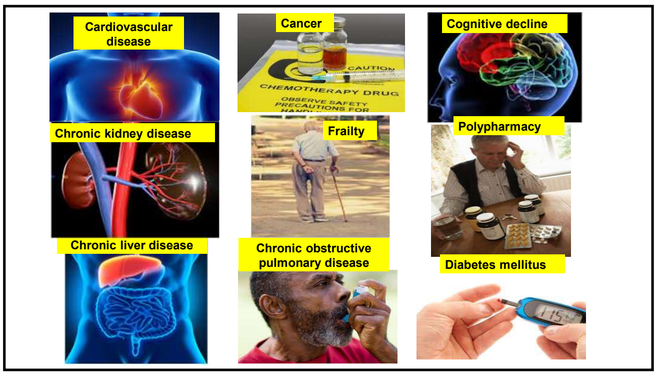
Cardiovascular disease
Cancer
Cognitive decline
Chronic kidney disease
Frailty
Polypharmacy
Chronic liver disease
Chronic obstructive pulmonary disease
Diabetes mellitus

7
